# MiR-124 suppresses cell motility and adhesion by targeting talin 1 in prostate cancer cells

**DOI:** 10.1186/s12935-015-0189-x

**Published:** 2015-05-06

**Authors:** Wei Zhang, Ye-qing Mao, Hua Wang, Wen-juan Yin, Shao-xing Zhu, Wei-cheng Wang

**Affiliations:** Department of Urology, Zhejiang Cancer Hospital, 38 Guangji Road, Hangzhou, Zhejiang 310000 China; Department of Urology, The First Affiliated Hospital of Zhejiang University, 79 Qinchun Road, Hangzhou, Zhejiang 310003 China; Department of Pathology, Zhejiang Cancer Hospital, 38 Guangji Road, Hangzhou, Zhejiang 310000 China; Medical equipment research institute of Zhejiang, 23 Huacheng Road, Hangzhou, Zhejiang 310000 China

**Keywords:** MiR-124, Talin 1, Prostate cancer, Migration, Invasion, Adhesion, Integrins

## Abstract

**Background:**

MicroRNA is a type of endogenous non-coding RNA implicated in various cellular processes, and has been intensely investigated in the field of cancer research for many years. Here, we investigated the functions and mechanisms of miR-124 in prostate cancer, which is a putative tumor suppressor reported in many carcinomas.

**Methods:**

Using bioinformatics, talin 1 was indicated as a potential target of miR-124. We examined the expression levels of miR-124 and talin 1 in tissue specimens and cell lines. To explore the relationship between miR-124 and talin 1, miR-124 mimics, miR-124 inhibitors, and talin 1 small interfering RNA (siRNA) were transiently transfected into cancer cell lines, followed by analysis using luciferase reporter assays. Next, to investigate the functions of miR-124 in prostate cancer, we performed cell attachment, migration, and invasion assays. A rescue experiment was also conducted to demonstrate whether miR-124 suppressed cell adhesion and motility by targeting talin 1. Finally, we examined the related signaling pathways of miR-124 and talin 1.

**Results:**

MiR-124 was down-regulated in prostate cancer specimens and cell lines, while talin 1 was over-expressed in prostate cancer specimens and cell lines. These results showed an inverse correlation of miR-124 and talin 1 expression. Similar to talin 1 siRNA, overexpression of miR-124 by transient transfection of mimics led to a significant decrease in talin 1 levels. Luciferase report assays showed that the seed sequence of the talin 1 3’-untranslated region was a target of miR-124. Functional investigations revealed anti-attachment, anti-migration, and invasion-promoting effects of miR-124 in prostate cancer cells. The rescue experiment confirmed that miR-124 exerted its biological functions by targeting talin 1. Finally, we found that miR-124 and talin 1 impaired cellular adhesion and motility through integrins and the focal adhesion kinase/Akt pathway.

**Conclusions:**

Our study demonstrated biological roles and the related mechanism of miR-124 in prostate cancer. The results indicate that talin 1 is very likely a novel player in the anti-metastatic signaling network of miR-124. By down-regulation of talin 1, miR-124 impairs the adhesion, migration, and invasion of prostate cancer cells.

## Background

In 1949, Huggins et al. revealed the androgen-driven trait of prostate cancer. Subsequently, hormone therapies were developed to treat this deadly male malignancy. Although the survival rate has been extended in the past few decades, complications such as metastasis still lead to the deaths of nearly 30,000 men per year. Curative treatments for men with metastatic lesions remain elusive [1].

MicroRNAs (miRNAs) are a class of small (19–25 nt) non-coding RNAs that play important roles in gene regulation by partial or full complementary matching with the 3′-untranslated region (UTR) of target mRNAs and triggering transcriptional or post-transcriptional suppression [2,3]. They are involved in numerous physiological functions such as cell differentiation, migration, proliferation, apoptosis, and senescence [4]. MiR-124, a putative tumor suppressor located in 8q12.3, is frequently found to be down-regulated in several human malignancies including bladder cancer, hepatocellular carcinoma, breast cancer, glioma, glioblastoma, cholangiocarcinoma, gastric cancer, osteosarcoma, ovarian cancer, and prostate cancer [5-17]. The biological effects of miR-124 in tumor cells are mainly negative regulation of cell proliferation, apoptosis, and especially metastasis. In particular, miR-124 is closely involved in tumor cell migration and invasion by targeting various molecules related to activation of the RhoA/ROCK pathway [6], matrix metalloproteinases (MMPs) [12,13,17], epithelial–mesenchymal transition [9,15], and even epigenetic modification [11], which are pivotal events in tumor metastasis.

Here, we identified a mechanism of the anti-metastasis effect of miR-124 in prostate cancer. By directly targeting talin 1, an integrin-associated cytoskeletal protein in focal adhesion formation, miR-124 suppresses the adhesion and motility of prostate cancer cells and down-regulates the focal adhesion kinase (FAK)/Akt pathway, suggesting the involvement of talin 1 in prostate cancer.

## Results

Inverse correlation between talin 1 and miR-124 levels in prostate cancer

**Figure 1:**
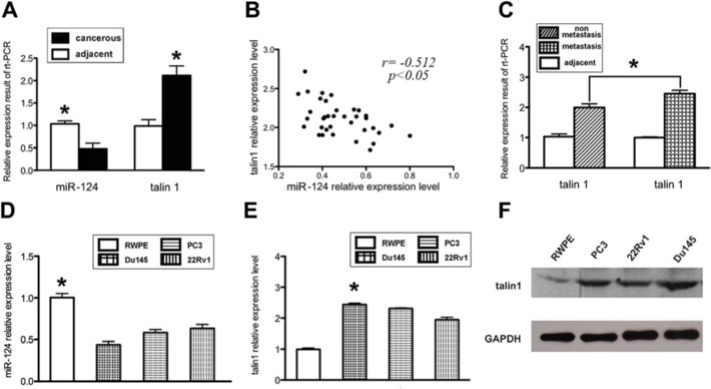
Expression levels of miR-124 and talin 1 in prostate cancer. **(A)** Quantification of miR-124 and talin 1 expression in surgical specimens from 37 prostate cancer patients by real-time PCR. The relative expression level of miR-124 in cancerous tissue was reduced by more than half of that in adjacent tissue, while the expression level of talin 1 in cancerous tissue was elevated by twice that in adjacent tissue. **(B)** Correlation analysis of miR-124 and talin 1 expression in the specimens demonstrated an inverse relationship (*r* = −0.512). **(C)** Comparison of talin 1 levels in the metastasis group (14 cases) and non-metastasis group (23 cases) revealed a relatively higher level of talin 1 in the metastasis group by approximately 1.25-fold. **(D)** Levels of miR-124 were examined in the prostate cancer cell lines, which showed a remarkably reduced level of miR-124 compared with the normal epithelial cell line. **(E)** Talin 1 levels were examined in prostate cancer cell lines, which showed remarkably elevated expression compared with the normal epithelial cell line. **(F)** Increased expression level of talin 1 in cell lines as shown by western blotting. Data represent the means from three independent experiments (*p < 0.05).

To investigate the expression of miR-124 in prostate cancer, its expression levels were examined quantitatively in 37 pairs of surgical specimens from prostate cancer patients by real-time PCR. In comparisons of the cancerous tissue and adjacent non-cancerous tissue in each pair, we found decreases of miR-124 expression at varying degrees in the cancerous tissues (>50% reductions, *p* < 0.05) (Figure 1A), which is consistent with previous reports [9,13]. Next, we examined the expression of talin 1. The results showed that talin 1 was universally over-expressed in cancerous tissue compared with the adjacent tissue (>2-fold increases, *p* < 0.05) (Figure 1A). The correlation analysis revealed an inverse relationship between miR-124 and talin 1 expression in these specimens (Figure 1B). Some studies have demonstrated that talin 1 is closely related to tumor metastasis [18,19]. Therefore, the surgical specimens were classified into a non-metastasis group (23 cases) and metastasis group (14 cases) according to the pathological results of lymph node biopsies. The metastasis group showed a modest increase in talin 1 expression compared with the non-metastasis group (~1.25 fold, *p* < 0.05) (Figure 1C). However, no differences in miR-124 expression were found between the two groups (data not shown).

Subsequently, miR-124 and talin 1 were examined and compared in a panel of prostate cancer cell lines and a normal prostate epithelial cell line, which showed similar results to the tissue specimens (*p* < 0.05) (Figure 1D–F).

These results not only confirmed up-regulation of talin 1 expression in prostate cancer, but also indicated an inverse correlation between miR-124 and talin 1 expression levels in prostate cancer, which implied a potential relationship.

### Talin 1 is a direct target of miR-124

**Figure 2:**
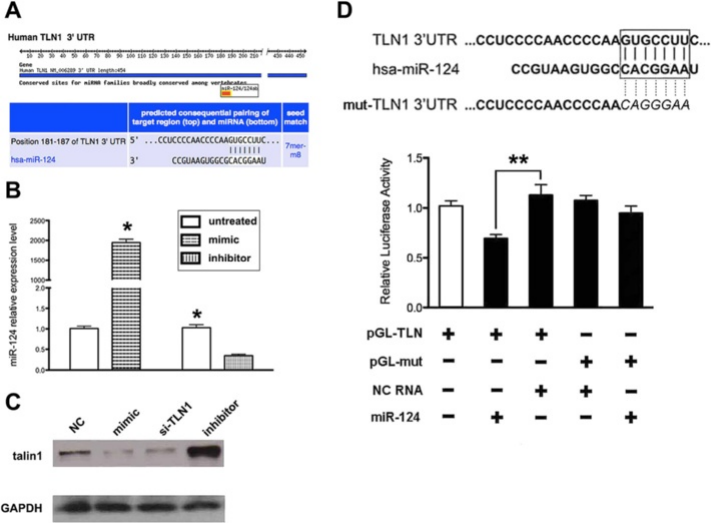
Talin 1 is a target of miR-124. **(A)** TargetScan revealed a putative interrelationship between talin 1 and miR-124. **(B)** To confirm transfection efficiencies, the level of miR-124 was examined after transfection of mimics or inhibitors. The miR-124 expression level was increased by approximately 2000-fold in cells transfected with mimics and decreased by 70% in cells transfected with inhibitors. **(C)** The expression level of talin 1 was obviously affected by manipulation of endogenous miR-124 in prostate cancer cells. **(D)** The dual luciferase reporter assay demonstrated regulation of talin 1 by miR-124. Relative light units were drastically suppressed by about 60% after transfection of pGL-TLN1 and mimics, implying an interaction between miR-124 and the seed sequence of the talin 1 gene. pGL-TLN1 denotes the reporter plasmid containing the seed sequence in the 3′-UTR of the talin gene. pGL-mut denotes the reporter plasmid with a mutated seed sequence. Data represent the means from three independent experiments (**p < 0.01).

To investigate the potential link between talin 1 and miR-124, we searched for putative target genes using TargetScan (http://www.targetscan.org/) and focused on talin 1 because of its rank and associations with cell migration and invasion (Figure 2A). Considering the significantly low level of miR-124 in the Du145 cancer cell line, we overexpressed and knocked down miR-124 in Du145 cells by transfection of miR-124 mimics or inhibitors, respectively (Figure 2B). To investigate the effects of miR-124 on talin 1 expression, we examined talin 1 levels after manipulation of miR-124 expression. Ectopic expression of miR-124 substantially decreased the expression of talin 1, which resembled knockdown of talin 1 by small interfering RNA (siRNA), whereas suppression of miR-124 by the inhibitors resulted in remarkable up-regulation of talin 1 (Figure 2C). Therefore, miR-124 acts to negatively regulate the expression of talin 1.

To confirm the relationship between miR-124 and talin 1, we performed a dual luciferase assay. HEK293T cells transfected with both the plasmid containing the target sequence in the talin 1 3′-UTR (pGL-TLN) and miR-124 mimics were defined as treatement group; the cells treated with the plasmid containing the mutated sequence (pGL-mut) and non-sense duplex sequence (NC RNA) were defined as controls. We found a specific interaction between exogenous miR-124 and the talin 1 3′-UTR, which led to suppression of luciferase activity (Figure 2D). These results demonstrated post-transcriptional regulation of talin 1 by miR-124. Therefore, talin 1 may be the novel target of miR-124.

### MiR-124 suppresses the motility and adhesion of prostate cancer cells *in vitro*

**Figure 3:**
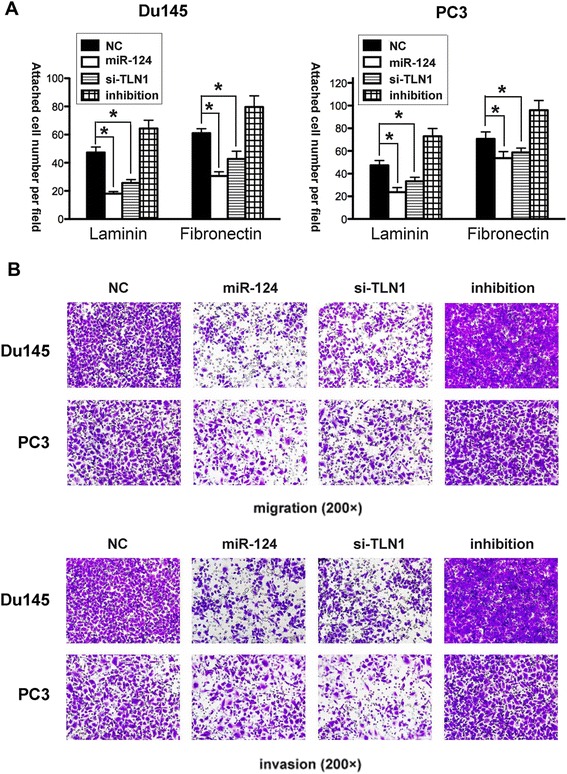
MiR-124 abrogates the adhesion, migration, and invasion capabilities of prostate cancer cells. Du145 and PC3 cells pre-transfected with mimics, inhibitors, or talin 1 siRNA were subjected to attachment and transwell assays. **(A)** MiR-124 mimics abrogated cell adhesion, resembling the effect of talin 1 knockdown, whereas miR-124 inhibitors relatively enhanced the adhesion of both cell lines. The effects were more prominent in Du145 cells. **(B)** Both mimics and talin 1 siRNA markedly impaired the migration and invasion capabilities of Du145 and PC3 cells, whereas miR-124 inhibitors had the opposite effects. Data represent the means from three independent experiments (*p < 0.05).

To functionally investigate the biological role of miR-124 in prostate cancer, we conducted gain-of-function experiments. Considering the implication of talin 1 in cell motility and adhesion, we performed transwell and attachment assays. The results showed that miR-124 transfectants not only exhibited a significant reduction in attachment to fibronectin, an essential component of the extracellular matrix (ECM) (*p* < 0.05) (Figure 3A), but also an impairment in cell migration and invasion capabilities (Figure 3B). These effects resembled those in cells with talin 1 knockdown by siRNA (*p* < 0.05). The results indicated that miR-124 might suppress the motility and adhesion of prostate cancer cells through talin 1.

Forced expression of talin 1 restores the miR-124-induced inhibition of cellular migration and invasion

**Figure 4:**
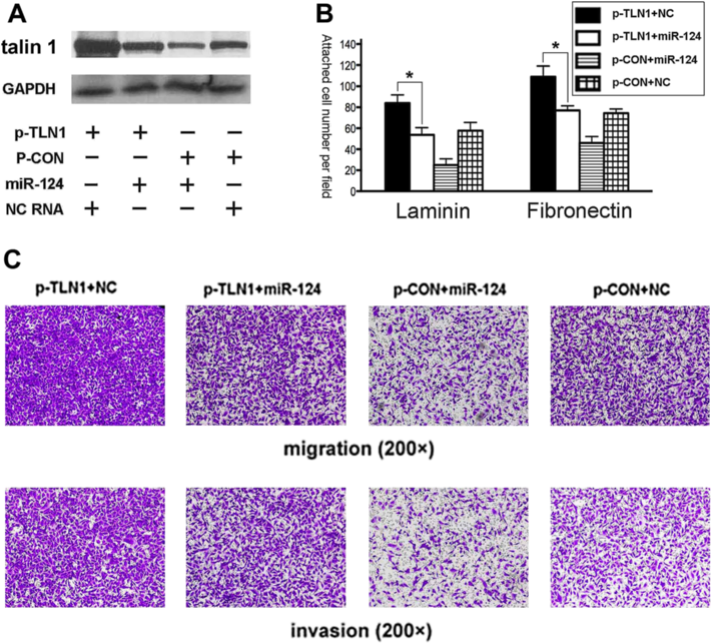
MiR-124 impairs cell adhesion and motility by targeting talin 1 directly. Du145 cells were co-transfected with miR-124 mimics or NC RNA and p-TLN or p-CON plasmids. **(A)** Western blot analysis of talin 1. The decreased level of talin 1 induced by miR-124 mimics could be restored by forced expression of talin 1. **(B)** Attachment and **(C)** Transwell assays demonstrated functional effects in cells after transfection. p-TLN denotes the expression plasmid containing the full-length coding sequence of talin 1. p-CON denotes the control plasmid containing the non-sense sequence. Data represent the means from three independent experiments (*p < 0.05).

To confirm regulation of talin 1 by miR-124 in cellular attachment, migration, and invasion, we ectopically expressed talin 1 by transfection of pCDNA3.1(-) containing the talin 1-coding sequence together with miR-124 mimics into Du145 cells. The elevated expression level of talin 1 was remarkably mitigated by forced expression of miR-124 (Figure 4A, lanes 1 and 2). Conversely, the decreased level of talin 1 by miR-124 mimics was significantly restored by forced expression of talin 1 (Figure 4A, lanes 2 and 3). Co-transfection of NC RNA and a non-sense plasmid (p-CON) was used for the control (Figure 4A, lane 4). Next, an attachment assay (Figure 4B) and transwell assay (Figure 4C) clearly demonstrated that talin 1 overexpression restored, to a certain extent, the abrogated attachment and motility of cancer cells induced by miR-124 mimics. Evidently, miR-124 targeted talin 1 directly, resulting in suppression of adhesion and motility in prostate cancer cells.

### Mechanistic investigation of the suppressive effect of miR-124 via talin 1

**Figure 5:**
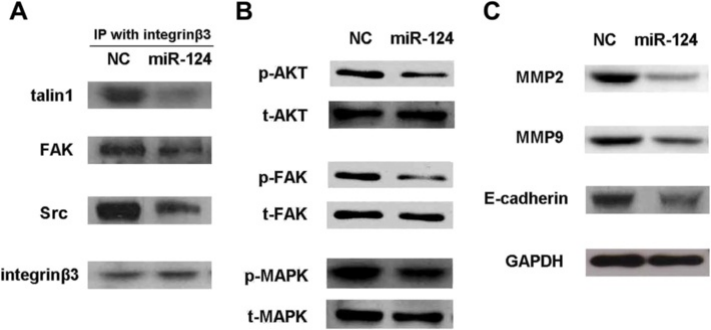
MiR-124 affects the downstream signaling pathway mediated by talin 1**.** DU-145 cells transfected with miR-124 mimics were subjected to immunoprecipitation using an anti-integrin β3 antibody. Talin 1, FAK, and Src binding was detected by their corresponding antibodies. **(A)** The results showed decreases in the binding of talin 1, Src, and FAK to integrin β3 after transfection of miR-124 mimics. **(B)** Levels of total and phosphorylated AKT (Ser473), FAK (Y397), and MAPK were down-regulated through suppression of the related signaling pathway by miR-124. **(C)** Levels of MMP2, MMP9, and E-cadherin proteins were decreased by transfection of miR-124 mimics.

We confirmed that talin 1 is a direct and functional target of miR-124 in prostate cancer. Next, we investigated the mechanism of tumor suppression by miR-124 via talin 1. Considering the varying levels of talin 1 among the prostate cancer cell lines, Du145 cells were selected for evaluation. Talin 1 acts as a mediator between integrins and the ECM through activation of integrins and recruitment of adaptor proteins. Recently, the FAK-src complex was identified as a pivotal partner in the interaction between talin 1 and the intracellular domains of integrins [20]. Aberrant activation of FAK-Src is common in tumorigenesis [21]. To better elucidate the suppressive role of miR-124 via talin 1, an immunoprecipitation assay was performed with an anti-integrin β3 antibody. The results showed a significant reduction in talin 1 binding to integrin β3, as well as FAK and Src after transfection of miR-124 (Figure 5A). Next, the total and phosphorylated protein levels of FAK, AKT, and MAPK were examined by western blotting in Du145 cells. The levels of phosphorylated FAK, Akt, and MAPK were decreased by miR-124, suggesting inhibition of the FAK/Akt pathway (Figure 5B). Additionally, invasion-related effectors MMP2, MMP9, and E-cadherin were down-regulated in the miR-124 transfectants, which might account for the impaired invasive and migratory capabilities of prostate cancer cells with miR-124 overexpression (Figure 5C).

## Discussion

As a type of adhesion receptor for ECM proteins, activated integrins promote tumor cell motility by forming focal adhesions [22]. Their activation requires adaptor proteins that facilitate interactions between integrins and actin fibers. Talin 1 is an important member of the adaptor protein family that plays an essential role in the activation of integrins. In mammals, there are two talin proteins encoded by TLN genes: ubiquitously expressed talin 1 and tissue-specific talin 2 [23]. An abnormal increase in the expression level of talin 1 has been recently reported in prostate cancer. Sakamoto et al. examined talin 1 expression in prostate cancer cells. They found that the levels of talin 1 correlated well with the malignant degree of the lesions. A higher talin 1 level corresponded to poorer differentiation and higher Gleason scores in surgically resected specimens [19]. Similarly, in our study, a remarkably higher level of talin 1 was detected in cancerous tissue in comparison with the adjacent tissue, as well as in the metastasis group compared with the non-metastasis group. These findings confirmed an abnormal expression signature of talin 1 in human prostate cancer.

The 270-kDa talin protein includes an N-terminal head domain, which is responsible for binding to the cytoplasmic tails of integrins, and a C-terminal flexible rod domain containing multiple binding sites for the F-actin-binding protein vinculin and a second integrin-binding site [24,25]. Functionally, direct binding of talin to the intracellular domain of an integrin destabilizes the transmembrane complex of the integrin, resulting in remodeling of the extracellular compartments and activation [26,27]. Simultaneous binding to actin filaments forms stable linkages between the ECM and cytoskeleton, leading to integrin clustering, aggregates of ECM proteins [24], assembly of actin filaments, and the formation of focal adhesions. These events increase the migratory capability of cells.

To the best of our knowledge, only one study has focused on talin 1 as a molecular target of miRNA. Tang et al. found that miR-9 inhibits tumor cell proliferation, migration, and invasion by suppression of talin 1 in ovarian serous carcinoma [28]. Here, we employed miRNA to specifically suppress talin 1 levels through binding to the 3′UTR of talin 1. We searched for potential miRNAs related to talin 1 using an online bioinformatics tool, and screened miRNAs by luciferase reporter assays in prostate cancer cells. The results suggested that miR-124 targeted the talin 1 3′-UTR by decreased levels of talin 1 in miR-124 transfectants.

For function investigations, we mainly focused on inhibition of cell adhesion and motility by miR-124 and talin 1. We simulated the extracellular environment of tumor cells by incubating the cells in fibronectin-coated dishes to examine their attachment capabilities. Similar to talin 1 knockdown, we found impaired adhesion of prostate cancer cells by over-expression of miR-124. In cell migration and invasion assays, significant reductions in the migration rates of cancer cells were observed in both miR-124 and talin 1 siRNA transfectants compared with the control group. Furthermore, a rescue experiment was performed to better illustrate the relationship between talin 1 and miR-124. It was clear that miR-124 counteracted the increase in talin 1 expression, resulting in mitigation of the enhanced cell adhesion and migration by talin 1. Interestingly, enforced expression of talin 1 could not sufficiently restore the effects of miR-124 on cell migration and invasion. We attribute this observation to other metastasis-related pathways of miR-124. Recent studies have shown that miR-124 mitigates tumor cell migration by directly targeting ROCK and Rac-1, both of which are components of the RhoA-ROCK cascade that regulates actomyosin contractility [6,12,29,30]. Thus, it is very likely that the enhanced migration of talin 1-overexpressing cells was mitigated by the anti-migration effect of miR-124 to some extent.

FAK binds to the cytoplasmic domain of integrins in part by association with adaptor proteins talin and paxillin. In turn, activated FAK forms the FAK-Src complex via an interaction with Src, and further promotes the phosphorylation of many related substrates that participate in reorganization of the actin cytoskeleton as well as cell migration [20]. The FAK/Akt signaling cascade acts as an essential downstream effector of integrin-focal adhesion interactions [19]. To reveal the mechanism, we performed an immunoprecipitation assay using an anti-integrin β3 antibody, and examined Akt, FAK, and MAPK, as well as their phosphorylated variants. The results showed that miR-124 and talin 1 might inactivate integrins and suppress the FAK/Akt pathway.

Src-FAK signaling has been recently reported to promote E-cadherin internalization, which facilitates tumor cell motility [31], inhibits the endocytic pathway, and activates MMP2 and MMP9 during cancer progression [32]. Accordingly, we identified suppression of effector proteins related to tumor invasion, MMP2, MMP9, and E-cadherin, in miR-124-transfected tumor cells. These results might illustrate how miR-124 confers non-migratory and non-invasive phenotypes to prostate cancer cells by targeting talin 1 in an integrin-dependent manner.

## Conclusions

Collectively, our study has shed light on the anti-migration and anti-invasion mechanisms of miR-124 in prostate cancer. Our preliminary experiments suggest that talin 1 is very likely a novel player in the anti-metastatic signaling network of miR-124, following ROCK, Rac-1, and transforming growth factor-α. Considering the limitations of *in vitro* experiments, further *in vivo* investigations are needed to confirm these results.

## Methods

### Clinical specimens

From 2013, 37 patients diagnosed with prostate adenocarcinoma underwent radical prostatectomy at the Department of Urology, Zhejiang Cancer Hospital. Lymph node metastasis was determined according to pathological analysis of biopsies obtained by lymphadenectomy. For each specimen pair, an experienced pathologist discriminated the cancerous nodule from the adjacent non-tumor tissue.

### Cell culture and transient transfection

Human prostate cancer cell lines PC3, Du145, and 22Rv-1, and the human prostate epithelial cell line RWPE were purchased from the Cell Bank of the Chinese Academy of Sciences (Shanghai, China). The human normal kidney cell line HEK293T was a kind gift by Dr Zhao An from the Central Laboratory of Zhejiang Cancer Hospital. All cells were maintained in RPMI-1640 medium (Invitrogen, Carlsbad, CA, USA) supplemented with 10% fetal bovine serum (FBS; Gibco, Grand Island, NY, USA).

After transfection of miRNA and/or siRNA, cells were harvested, counted, and seeded into six-well plates (Costar, Corning, CA, USA). Lipofectamine 2000™ reagent (Invitrogen) was employed to transfect siRNA (GenePharma, Shanghai, China) miR-124 mimics (RiboBio, Guangzhou, China), and miR-124 inhibitors (RiboBio, Guangzhou, China) into cells at 50, 100, and 200 nM, respectively. For mimics, NC RNA (the negative control), inhibitors, and siRNA, the duration of transfection was 48 h. For co-transfection with plasmids, transfection was performed for 24 h. The sequences were as follows (5′–3′): NC RNA, ACUACUGAGUGACAGUAGA; has-miR-124 [Pubmed Nucleotide: accession number: MI0000443], GGCAUUCACCUCGUGCCUUA; has-miR-124 inhibitors, UAAGGCACGCGGUGAAUGCC; talin 1 siRNA, GAAGCCUCUUCUAUUUAAUGCAGAC.

### 3′-UTR vector construction for luciferase reporter assays

The talin 1 3’-UTR fragment containing the seed sequence was amplified by PCR using cDNA from RWPE cells and the following primers: forward, 5′-CGAGCTCCAGTCCCGCAGTACAT-3′; reverse, 5′-GCCGCGGTGGGGGAAGATAGTAT-3′. The amplified fragment was cloned downstream of the luciferase-coding sequence in a pmir-GLO expression vector (Promega, Wisconsin, USA) at the sites of Sal I and Sac I endonucleases (Takara, Dalian, China). The vector containing the seed sequence was called pGL-TLN1. A control vector containing a mutated sequence generated by a quickChange™ Site-directed Mutagenesis kit (Agilent Technologies, Santa Clara, CA, USA) was called pGL-mut. HEK293T cells were transfected with 100 ng pGL-TLN1 + NC RNA, pGL-TLN1 + miR-124, pGL-mut + miR-124, and pGL-mut + NC RNA. After 24 h, the cells were harvested and subjected to a Dual Luciferase Reporter Assay kit (Promega, Wisconsin, USA). The lysate was then analyzed by a bioluminescence detection system (Berthold Technologies, Bad Wildbad, Germany) to determine the relative light units.

### Transwell migration and invasion assays

After transfection, 1 × 10^5^ cells suspended in 200 μL RPMI-1640 medium without FBS were added to the upper chamber of a Millicel transwell chamber (8-μm pore size, Millipore, Billerica, USA). For invasion assays, the filter membrane was coated with 30 μL matrigel (BD Biosciences, Franklin Lakes, USA) diluted in RPMI-1640 medium at a ratio of 1:8. A total of 600 μL medium containing 10% FBS was added to the lower chamber. After incubation for 24 h, the migrated/invaded cells were fixed with methanol, stained with 1% crystal violet, and counted under an inverted microscope.

### Attachment assay

After transfection, cells were collected, counted, and seeded in 24-well plates (1 × 10^5^ cells per well) coated with fibronectin (BD Biosciences, Franklin Lakes, USA). After 1 h of incubation, the unattached cells were removed by washing with PBS three times. The remaining cells were fixed with methanol, stained with 1% crystal violet, and counted under an inverted microscope.

### Real-time RT-PCR

To determine the relative expression levels of miR-124 and talin 1, total RNA and small RNA were extracted from cells using RNAiso Plus reagent and RNAiso for Small RNA reagent (Takara), respectively. cDNA of miRNA was then synthesized using a One Step PrimeScript® miRNA cDNA Synthesis Kit. Total cDNA was synthesized using a PrimeScript® RT reagent Kit (Takara). Subsequently, real-time RT-PCR was performed with SYBR® Premix Ex Taq™ II (Takara) on an ABI 7500 Real-time PCR System (Applied Biosystems, Foster City, CA, USA). U6 and glyceraldehyde 3-phosphate dehydrogenase (GAPDH) genes were used as endogenous controls. The primers were as follows: miR-124, 5′-TAAGGCACGCGGTGAATGCC-3′; U6, 5′-TGCGGGTGCTCGCTTCGGCAGC-3′; talin 1 forward, 5′-TGAGTCAGTGTGCCAAGAA-3′ and reverse, 5′-TAGATTCTGTACCACACT-3′; GAPDH forward, 5′-AAGGTGAAGGTCGGAGTCA-3′ and reverse, 5′-GAAGATGGTGATGGGATTT-3’. The relative expression levels of target genes were quantified by normalization to endogenous U6 or GAPDH expression levels, which was calculated as 2-^ΔΔ^C(t).

### Immunoprecipitation

Immunoprecipitation was performed with a Pierce Classic IP Kit (Thermo Scientific, Austin, TX, USA). DU-145 cells as well as NC RNA and miR-124 transfectants were lysed in RIPA buffer (Beyotime Biotech, Hangzhou, China). Total protein was extracted and quantified. Protein samples (100 μg) were incubated overnight at 4°C with the antibody. After incubation of the lysates with Protein G Plus/Protein A agarose beads (30 μL) for 1 h at 4°C, the agarose was centrifuged in PBS and then subjected to SDS-polyacrylamide gel electrophoresis (PAGE). Finally, the proteins were transferred to 0.22-μm nitrocellulose membranes (Abcam, Cambridge, MA, USA).

### Western blot analysis

Total proteins in cells were extracted using RIPA buffer, quantified using a Pierce BCA Protein Assay Kit (Thermo Scientific, Austin, TX, USA), separated on 6% SDS-PAGE gels, and then transferred onto nitrocellulose membranes. The membranes were blocked in 5% dry skim milk for 1 h at room temperature, followed by incubation with each primary antibody overnight at 4°C. Rabbit or mouse monoclonal antibodies against talin 1 were obtained from Abcam. Antibodies against FAK, Akt, phospho-FAK (Y^397^), phospho-Akt (S^473^), MAPK, and phospho-MAPK were purchased from Cell Signaling Technology (Beverly, MA, USA). Antibodies against integrin β3, Src, MMP2, MMP9, E-cadherin, and GAPDH were from Epitomics (Burlingame, CA, USA). The membranes were then incubated with secondary antibodies and visualized using an EZ-ECL kit HRP (BioInd, Kibbutz Beit Haemek, Israel).

### Over-expression vector construction and rescue experiment

To investigate the effect of talin 1 over-expression in cells, we designed a pair of primers (forward: 5′-CCGGATCCATGGTTGCACTTTCACT-3′; reverse: 5′-CCGAATTCTAGAAGAGGCTTCTTT-3′) to amplify the coding sequence of the talin 1 gene using cDNA from RWPE cells by PCR. The amplified sequence was then cloned into a pCDNA3.1+ expression vector (Invitrogen) using BamHI and EcoRI endonucleases (Takara). The vector containing the target sequence was called p-TLN1. A control vector with a mutated sequence generated by the quickChange™ Site-Directed Mutagenesis kit was called p-CON. To directly examine the interaction between miR-124 and talin 1 in prostate cancer cells, we performed a rescue experiment. The over-expression vector was transfected with Lipofectamine 2000™ reagent at 1 μg per well. The cells were co-transfected with p-TLN1 + NC RNA, p-TLN1 + miR-124, p-CON + miR-124, and p-CON + NC RNA for 24 h and then harvested for western blot and functional analyses.

### Statistical analysis

The Student’s t-test was performed using SPSS version 17.0 software for statistical analysis. A *p*-value of less than 0.05 was considered statistically significant. Data represent the means ± standard deviation (standard deviation) of three independent measurements. The correlation analysis was performed and charted by the software Prism Graphpad version 5.0c.
